# Dietary intake of tyrosine and phenylalanine, and p-cresyl sulfate plasma levels in non-dialyzed patients with chronic kidney disease

**DOI:** 10.1590/2175-8239-JBN-2018-0214

**Published:** 2020-05-18

**Authors:** Andressa Louzada Frauche Fernandes, Natalia A. Borges, Ana Paula Black, Juliana dos Anjos, Greicielle Santos da Silva, Lia S. Nakao, Denise Mafra

**Affiliations:** 1 Universidade Federal Fluminense, Programa de Pós-Graduação em Ciências da Nutrição, Niterói, RJ, Brasil.; 2 Universidade Federal Fluminense, Programa de Pós-Graduação em Ciências Cardiovasculares, Niterói, RJ, Brasil.; 3 Universidade Federal Fluminense, Programa de Pós-Graduação em Ciências Médicas, Niterói, RJ, Brasil.; 4 Universidade Federal do Paraná, Departamento de Patologia Básica, Curitiba, PR, Brasil.

**Keywords:** Renal Insufficiency, Chronic, Diet, Food, and Nutrition, Gastrointestinal Microbiome, Cardiovascular Diseases, Insuficiência Renal Crônica, Alimentos, Dieta e Nutrição, Microbioma Gastrointestinal, Doenças Cardiovasculares

## Abstract

**Background:**

Patients with chronic kidney disease (CKD) present an imbalance of the gut microbiota composition, leading to increased production of uremic toxins like p-cresyl sulfate (PCS), product from bacterial fermentation of the amino acids tyrosine (Tyr) and phenylalanine (Phe) from the diet. Thus, diet may be a determinant in the uremic toxins levels produced by the gut microbiota. The aim of this study was to evaluate the possible relationship between Tyr and Phe intake and PCS plasma levels in non-dialysis CKD patients.

**Methods:**

Twenty-seven non-dialysis CKD patients (stages 3 and 4) without previous nutritional intervention were evaluated. The dietary intake was evaluated using a 24-hour recall, 3-day food record and protein intake was also estimated by Protein Nitrogen Appearance (PNA). The plasma levels of PCS were measured using reverse phase high performance liquid chromatography.

**Results:**

The evaluated patients (GRF, 34.8 ± 12.4 mL/min, 54.2 ± 14.3 years, BMI, 29.3 ± 6.1 kg/m^2^) presented mean protein intake of 1.1 ± 0.5 g/kg/day), Tyr of 4.5 ± 2.4 g/day and Phe of 4.6 ± 2.5 g/day. PCS plasma levels (20.4 ± 15.5 mg/L) were elevated and positively associated with both, Tyr (r = 0.58, *p* = 0.002) and Phe intake (r = 0.53, *p* = 0.005), even after adjustments for eGFR and age.

**Conclusion:**

This study suggests that the diet is an important modulator of the uremic toxins plasma levels produced by the gut microbiota, in non-dialysis CKD patients.

## RESUMO

**Introdução:**

Pacientes com doença renal crônica (DRC) apresentam desequilíbrio na composição da microbiota intestinal, gerando toxinas urêmicas, como o p-cresil sulfato (PCS), pela fermentação bacteriana dos aminoácidos tirosina (Tyr) e fenilalanina (Phe) da dieta. Assim, a dieta pode ser determinante nos níveis de toxinas urêmicas produzidos pela microbiota intestinal. O objetivo deste estudo foi avaliar a possível relação entre a ingestão de Tyr e Phe e os níveis plasmáticos de PCS em pacientes com DRC não dialisados.

**Métodos:**

Foram avaliados 27 pacientes com DRC em tratamento conservador (estágios 3 e 4), sem intervenção nutricional prévia. A ingestão alimentar foi avaliada pelo recordatório alimentar de 24h (R-24h) de 3 dias, e a ingestão proteica também foi verificada através do Protein Nitrogen Appearance (PNA). Os níveis plasmáticos de PCS foram determinados por cromatografia líquida de fase reversa.

**Resultados:**

Os pacientes avaliados (TFG, 34,8 ± 12,4 mL/min, 54,2 ± 14,3 anos, IMC 29,3 ± 6,1 kg/m^2^) apresentaram ingestão média de proteína de 1,1 ± 0,5 g/kg/dia, Tyr de 4,5 ± 2,4 g/dia e Phe de 4,6 ± 2,5 g/dia. Os níveis plasmáticos de PCS (20,4 ± 15,5 mg/L) foram elevados e positivamente associados à ingestão de Tyr (r = 0,58, *p* = 0,002) e Phe (r = 0,53, *p* = 0,005), mesmo após ajustes pela TFG e idade.

**Conclusão:**

Este estudo sugere que a dieta é um importante modulador dos níveis plasmáticos de toxinas urêmicas produzidas pela microbiota intestinal em pacientes com DRC não dialisados.

## INTRODUCTION

With chronic kidney disease (CKD) progressing, an increasing inability to maintain homeostasis and excrete metabolism products exposes these patients to a high risk of death from Cardiovascular Diseases (CVD).^1^^-^ 3 Among the many factors involved in the progression of CKD and CVD pathology are the high levels of urinary toxins, resulting from changes in the profile and behavior of the intestinal microbiota and the in the kidneys’ inability to properly clear these metabolites.^4^^-^ 8

Nutrient availability is a key factor in modulating microbial heterogeneity and activity, which may compromise the intestinal microbiota balance and change the profile of bacterial metabolites.^6^ Furthermore, metabolites of the intestinal microbiota can alter the luminal pH, the intestinal wall integrity and interfere in the host’s homeostasis.^5^^,^ 9 ^-^ 10 Among the bacterial phyla that inhabit or intestine, Firmicutes and Bacteroidetes are the predominant ones; therefore, their balance is essential to maintain the proper interaction between the intestinal microbiota and the host.^2^^,^ 11 ^-^ 12

The bacteria that make up the intestinal microbiota have several functions, such as produce energy, degrade polysaccharides and extracellular amino acids using hydrolases, polysaccharidases and deaminases, generating products that act positively or negatively on the bodies of CKD patients.^2^^,^ 10 ^,^ 13 Such bacteria use prebiotic dietary fibers as a substrate for making short-chain fatty acids (AGCC), which help maintain the colonocytes and the immune response^14^; on the other hand, the fermentation of diet-borne protein generates metabolites known as uremic toxins.^15^^-^ 16

Upon reaching the large intestine, dietary proteins and peptides in the diet undergo depolymerization by proteases and peptidases from bacteria, generating small oligopeptides and amino acids that are available for assimilation by the colon microbiota. Predominantly distal part of the colon, the aromatic amino acids tyrosine (Tyr) and phenylalanine (Phe) are converted by bacterial fermentation into compounds such as phenol and p-cresol, through a series of deamination, transamination and decarboxylation tests. In the liver, p-cresol is sulfated and transformed into p-Cresyl Sulfate (PCS).^17^ This toxin has an important metabolic role, and studies show a positive relationship between PCS levels and cardiovascular events in patients with CKD.^10^^,^ 18 ^-^ 19 Due to the high PCS levels seen in CKD patients and their relationship with negative effects, we need to assess the dietary intake of Tyr and Phe in patients with CKD under conservative treatment and its Influence on the plasma levels of this uremic toxin.

## MATERIAL AND METHODS

### STUDY POPULATION

We ran a cross-sectional study involving 27 patients with CKD in stages 3 and 4 who sought care at the Renal Nutrition Clinic of the Faculty of Nutrition of the Universidade Federal Fluminense (UFF) and who were included in the study by our research group^20^.

We excluded patients with AIDS, cancer, autoimmune diseases, inflammatory diseases, liver disease or smoking, as well as patients who used drugs, probiotics, symbols or antibiotics in the last 3 months. We included patients aged over 18 years, with CKD levels between 3 and 4 and without prior nutritional counseling. The Research Ethics Committee of the School of Medicine of Universidade Federal Fluminense approved the research protocol under number 26698914.7.0000.5243 and all patients signed the free and informed consent form.

### FOOD INTAKEASSESSMENT

A nutritionist estimated the participants’ food intake using the 24-hour Food Record (R24h) of three different days, including a weekend day. To quantify the dietary intake of proteins, total fibers, Tyr and Phe, we gathered the data captured in the R24h in a Microsoft Office Excel (2007) spreadsheet, and we calculated the variables of interest based on the National Food Composition Table Agricultural Library version 3.9.5.1^21^ and the Brazilian Food Composition Table.^22^ In addition, the protein intake was also estimated through the Protein Nitrogen Appearance (PNA),^23^ corrected by the current weight, according to the formulas described in Chart 1.^23^ We added the Tyr and Phe intake, and the result was normalized by bodyweight to make it possible to compare with a recommendation from the Institute of Medicine, which does not bring the recommended intake values for each of these amino acids.^24^

**Chart 1:** Equations for estimating PNA during the non-dialysis phase of CKD

Urinary Nitrogen Calculation	Protein Intake Calculation
1) UUN = UV x (UU ÷ 2.14)	2) PNA (g ptn/day) = [UUN + (0.0031g N x kg)] x 6.25

UUN: urinary urea nitrogen; UV: urinary volume of 24 h (L); UU: urinary urea (g/L);

PNA: protein ingested g/day; N: nitrogen.

Source: National Kidney Foundation.^23^

### NUTRITIONAL STATUS ASSESSMENT AND BODY COMPOSITION

We assessed the patients’ nutritional status using the following anthropometric parameters: body weight (kg), measured on a calibrated FILIZOLA^®^ scale, with a maximum capacity of 150 kg and precision of 0.1 kg. The individuals were instructed to position themselves in the center of the scale’s base, with their feet close together, bare, with light clothes and arms joined to the sides of the body; their height (m) was measured with the aid of a stadiometer attached to the aforementioned scale, with the individual standing tall, barefoot, with the arms close to the sides of the body and with the eyes fixed on the horizon. With this information at hand, we calculated their body mass index (BMI) by the ratio between current body weight (kg) and height (m) squared (kg/m^2^) and their nutritional status was established based on definitions proposed by the World Health Organization.^25^

The total body fat percentage (% fat) and total lean body mass (% lean mass) were measured by a dual energy X-ray absorptiometry - DXA, Lunar Prodigy Advance Plus model, from General Electric Madison, Wisconsin, USA. The analyses were performed at the UFF Nutritional Assessment Laboratory (LANUFF) and the values obtained were compared with the parameters from Lohman et al.^26^

### DETERMINATION OF ROUTINE BIOCHEMICAL PARAMETERS

We measured routine biochemical parameters, such as blood glucose, creatinine, urea, uric acid, total cholesterol, HDL, triglycerides, albumin, sodium, potassium and phosphorus using Bioclin^®^ kits (Bioclin BS-120 Chemistry Analyzer) from the UFF (LABNE) Experimental Nutrition Laboratory. The LDL-c values were obtained using the Friedewald et al.^27^ equation. The glomerular filtration rate (GFR) was estimated using the CKD-Epi equation.^28^ All biochemical parameters were classified according to Merck Sharp & Dohme Corp references^29^ and those from the Brazilian Society of Cardiology.^30^

### PCS PLASMA LEVELS DETERMINATION

We determined total PCS plasma levels using the Reverse Phase High Performance Liquid Chromatography (RP-HPLC, Shimadzu, Zellik, Belgium) connected to the fluorescence detector, as described by Borges et al.^31^ We compared the mean plasma PCS values obtained with the references of the European Uremic Solutes Database (EuTox).^32^

### STATISTICAL ANALYSIS

We used the Kolmogorov-Smirnov test to assess the distribution of variables. The results were expressed as mean ± standard deviation (SD) or median (interquartile range). We assessed the correlations between variables using the Spearman Rho correlation, or the Pearson’s coefficient, depending on the sample distribution. We ran a multivariate analysis to assess factors associated with uremic toxin levels. Statistical significance was accepted as *p* ≤ 0.05 and the analyses were performed using the SPSS Statistics for Windows software, version 23.0 (SPSS, Inc., Chicago, IL).

## RESULTS

The age of the patients studied ranged from 29 to 77 years, and 48% were males. According to the GFR, 63% of patients were in stage 3 of CKD, 29.6% in stage 3a and 33.4% in stage 3b, while 37% were classified in stage 4 of the disease. The main comorbidities were hypertension (96.3%), followed by dyslipidemia (37%) and diabetes mellitus (29.6%). Table 1 depicts the patients` anthropometric and demographic characteristics.

**Table 1:** Anthropometric and demographic characteristics of patients in stages 3 and 4 of CKD

Variables	Values
Age (years)	54,2 ± 14,3
Weight (kg)	78,2 ± 18,2
Height (m)	1,6 ± 0,1
Body Mass Index (kg/m^2^)	29,3 ± 6,1
% Body Fat	36,3 ± 8,0
% Lean Mass	59,0 ± 9,0

Results shown as Mean ± SD.

According to the BMI, 44% of the patients were obese (BMI > 30 kg/m^2^), 26% were overweight, 26% were eutrophic and only one patient was thin. Insofar as body fat percentage is concerned, 89% of the patients (48% women and 41% men) had high values, according to the reference table.^26^

Table 2 shows routine biochemical parameters and plasma concentrations of PCS uremic toxin. Average plasma concentrations of total PCS were high compared to normal mean values (individuals without CKD) presented in the EUTox database (1.87 mg/L ± 2.31 mg/L).^32^

**Table 2:** Routine biochemical parameters and mean PCS and GFR levels of patients in CKD stages 3 and 4

Variables	Values
Glucose (mg/dL)	106,5 ± 54,3
Creatinine (mg/dL)	2,2 ± 0,9
Urea (mg/dL)	71,3 ± 28,4
GFR (ml/min/1,73m²)	34,8 ± 12,4
Uric acid (mg/dL)	6,4 ± 1,3
Total cholesterol (mg/dL)	184,0 ± 46,0
HDL (mg/dL)	52,1 ± 14,4
Triglycerides (mg/dL)	146,5 ± 62,2
LDL (mg/dL)	102,7 ± 38,2
VLDL (mg/dL)	29,3 ± 12,4
Albumin (g/dL)	3,7 ± 0,3
Potassium (mmol/L)	4,3 ± 0,6
Phosphorus (mg/dL)	3,5 ± 1,3
PCS (mg/L)	20,4 ± 15,5

GFR: glomerular filtration rate; LDL: Low-density lipoprotein; HDL: High-density lipoprotein; VLDL: very Low-density lipoprotein; PCS: p-cresyl sulfate. Results presented as mean ± SD.

Table 3 depicts protein intake values, as well as total fiber, Tyr and Phe, obtained by R24h, and PNA values. The protein intake found by both R24h and PNA was above the recommendations for patients undergoing conservative treatment^33^ (it should be noted that these patients had not yet received a prescription for a hypoprotein diet). Total fiber intake was also below recommended values.^34^ The daily intake of Tyr + Phe was higher than the recommendations for healthy individuals according to Dietary References.^24^

**Table 3:** Food intake of proteins, total fibers, Tyr and Phe of patients in stages 3 and 4 of CKD

Variables	Mean	Recommendation
PNA (g/kg/day)	1.0 ± 0.4	0.6 - 0.8
Protein (g/kg/day)	1.1 ± 0.5	0.6 - 0.8^a^
Fiber (g/day)	22.4 ± 8.1	25 - 30^b^
Tyr (g/day)	4.5 ± 2.4	-
Phe (g/day)	4.6 ± 2.5	-
Total Tyr + Phe (g/kg/day)	0.12 ± 0.06	0.033g/kg/day^c^

Results presented as Mean ± SD.

a KDOQI^33^

b American Dietetic Association^34^

c Institute of Medicine^24^

**Figure 1:**
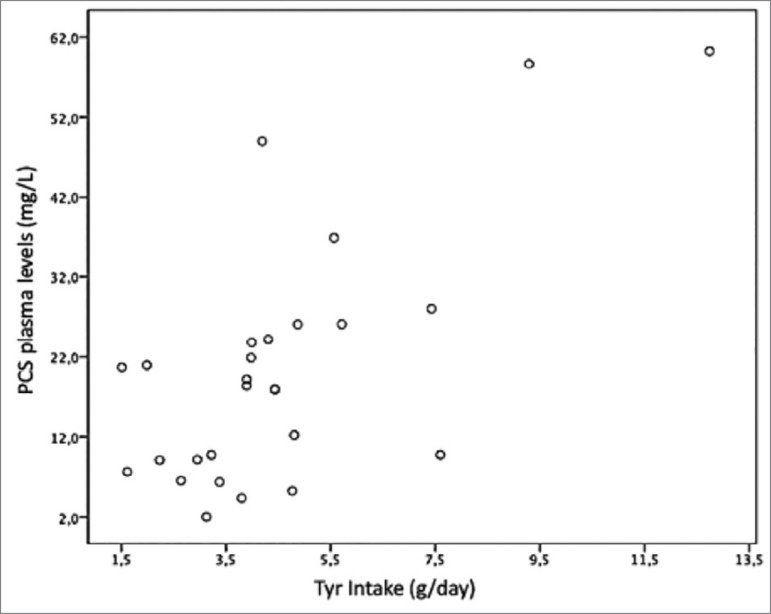
Correlation between plasma PCS levels and Tyr intake (r = 0.58; *p* = 0.002).

**Figure 2:**
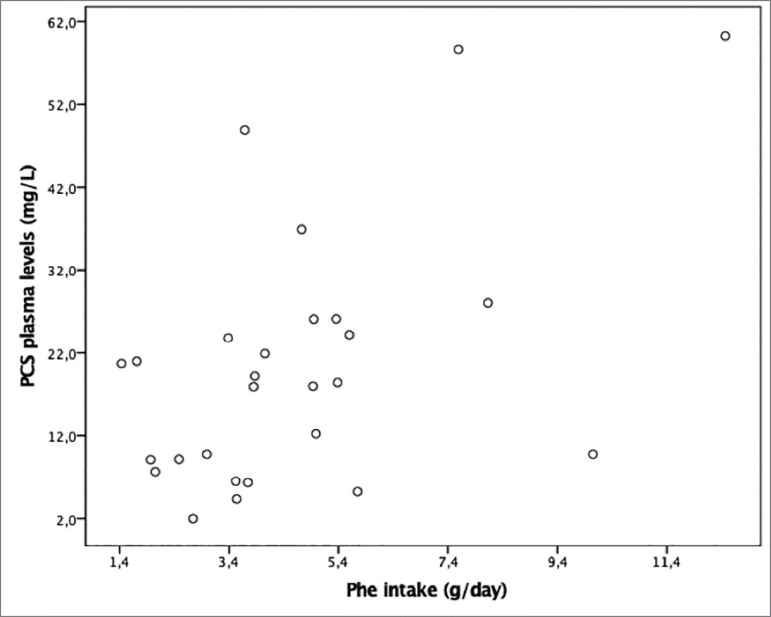
Correlation between PCS plasma levels and Phe intake (r = 0.53; *p* = 0.005).

There was a positive correlation between plasma PCS levels and Tyr intake (r = 0.58, *p* = 0.002) (Figure 1) and Phe (r = 0.53, *p* = 0.005) (Figure 2), which remained even after adjusting for GFR and age (Table 4). It is noteworthy that the plasma levels of these toxins increased with the renal failure progression.

**Table 4:** Multivariate analysis of the association between possible predictors of plasma PCS levels

Variables	β-coefficient	*p* -value
Age	-0,02	0,89
GFR	-0,36	0,008
Tyr	0,52	0,001
Phe	0,39	0,007

GFR: glomerular filtration rate; Tyr: tyrosine; Phe: phenylalanine.

## DISCUSSION

We found a positive correlation between Tyr and Phe intake and PCS plasma levels in non-dialyzed CKD patients. We also found daily intake higher than the dietary recommendations of these amino acids among healthy individuals, both by the R24h method and PNA.^24^ These findings help explain the relationship between food intake and uremic toxins production by the intestinal microbiota, which therefore reinforces the importance of implementing nutritional strategies to delay the progression of CKD and cardiovascular adverse outcome, since uremic toxins are important predictors of such events.

On the other hand, Brito et al. (2016) found no association between the levels of indoxyl sulfate and the intake of its precursor, the amino acid tryptophan, in hemodialysis patients. However, the authors noted that the protein intake was below the recommended values for these patients, and the tryptophan intake was close to the recommendations in the Dietary Reference Intakes (DRI),^35^ differently from the patients in the present study, who presented high protein intake (for patients with CKD under conservative treatment) and the investigated amino acids. Toden et al. (2005) reported an increase in the production of uremic toxins, including PCS, in rats fed a high-protein diet.^36^

It is worth mentioning that several studies show that PCS participates in worsening renal function, increased inflammation and oxidative stress in patients with CKD. Liabeuf et al. (2010) found high serum PCS levels in 139 individuals in the last stages of CKD (especially from stage 4) and pointed out that the relationship between PCS and mortality was independent of other etiological causes.^37^ PCS has also been associated with CKD progression^38^ and, in addition, it correlates with increased vascular stiffness,^39^ cardiovascular disease and mortality in individuals in stage 5 CKD.^40^

From stage 3 onwards, the substantial impairment of renal functions causes the plasma retention of several metabolites, establishing the so-called uremic syndrome or uremia.^6^ These compounds are classified as toxins because they present high serum concentrations, establishing deleterious interactions over a series of organic activities.^1^ For the production of PCS, anaerobic bacteria must ferment the Tyr and Phe amino acids,^2^ from protein dietary sources such as meat, chicken, cheese, eggs and milk.^21^ In the intestine, microorganisms convert them to 4-hydroxyphenylacetic acid and subsequently p-cresol,^1^ which is sulfated in the submucosal layer, resulting in PCS.^2^^,^ 15 ^-^ 17 Its presence in blood circulation stimulates the inflammatory response by activating leukocytes, releasing cytokines, producing reactive oxygen species (ROS), oxidative stress and damage to the endothelium, promoting atherosclerosis.^4^^,^ 9

In addition, the high plasma urea concentration detected among the participants of this study (as expected for patients in the stages of CKD they were in) is responsible for the increase in urea concentrations in the intestinal lumen, altering the biochemical environment and contributing to growth of bacteria species that are more adapted to this substrate; thus being one of the factors that contribute to dysbiosis and the increase in the synthesis of uremic toxins in CKD.^10^ Vaziri et al.,^41^ analyzed this interaction between uremia and intestinal changes in order to determine whether the products of urea degradation in the intestine impacted the mucosal integrity. In fact, there were changes in the intestinal barrier cells, and these findings help to clarify the factors that trigger disturbances that make it more permeable to the diffusion of toxins from the intestinal lumen to the blood current.^9^^,^ 41

An important characteristic of nutritional therapy during the non-dialysis phase is the adoption of a hypoprotein diet, which consequently reduces the supply of nitrogen compounds to the intestine.^2^ Black et al.^20^ investigated the influence of the hypoprotein diet (0.6 g/kg/d) on the microbial profile and plasma levels of uremic toxins in patients with CKD undergoing conservative treatment, and found a significant reduction in PCS levels in the group of patients who complied with the hypoprotein diet, in addition to changes in the profile of their intestinal microbiota. In another study, Marzocco et al. (2013) showed that the prescription of a protein-restricted diet (0.3 g/kg/d, supplemented with keto analogs and essential amino acids), reduced the levels of indoxyl sulfate in patients with CKD under conservative treatment.^42^ However, such studies have not evaluated the intake of the precursor amino acids of uremic toxins.

Additionally, another approach that can help CKD patients is the supply of dietary fibers, an important substrate for the symbiotic colonic bacteria.^43^ Its relevance was pointed out by Dominianni et al.,^44^ when carrying out the genetic sequencing of the microbiota of 82 individuals and notedthe that fiber intake modulated the bacterial profile. In the case of the participants in this study, fiber intake was below recommendations, impairing its availability as a substrate for intestinal bacteria. In addition to the influence of Western eating habits,^45^ fiber intake in these patients may be compromised due to the control of hyperkalemia, since potassium is present in high amounts in fruits and vegetables, which are also important sources of fiber.^19^^,^ 46 It is worth mentioning that, regardless of this restriction, the patients in the present study had not, until now, received nutritional guidance, which may justify the quantitative and qualitatively inadequate intake in the face of CKD.

Given the above, it is interesting to note that dietary intervention in CKD, targeting intestinal health, can play a relevant role in the homeostasis of these patients, as well as in the development of comorbidities. In this context, Moradi et al.^47^ emphasize the relevance of adjuvant treatments that focus on exogenous sources of uremic toxin precursors, in order to minimize their production by colonic bacteria, with the objective of reducing the exposure of these patients to cardiovascular diseases.

This study encountered some limitations during its execution. First, we obtained the data from a small population sample of CKD patients, lacking a more significant number to strengthen the present findings. Second, regarding R24h, the accuracy in obtaining the data depends on the interviewee’s memory. To overcome this limitation, we used the PNA technique to measure protein intake more accurately. Despite this, R24h is a subjective method validated and widely used in studies related to food intake.^48^ Thirdly, because a cross-sectional study was performed, the plasma levels of PCS were measured only once, without exploring the intra-individual variables of the participants. Finally, observational analysis does not allow modulating food intake to more precisely identify the effects of dietary variables on the production of uremic toxins. In addition, there is a scarcity of studies on the intake of Tyr and Phe and their relationship with plasma PCS levels, requiring further studies to be conducted.

## CONCLUSION

The results of this study showed that the food intake of Tyr and Phe may represent an important factor capable of influencing the plasma levels of PCS, highlighting the role of one’s diet in the modulation of metabolites from the intestinal microbiota, and suggesting that, in addition to the quantitative control of protein intake in the conservative treatment of CKD, attention should also be given to the amino acid profile of the diet. Thus, the relevance of specific nutritional interventions for these patients is emphasized as an essential component of their treatment, in order to promote better quality of life and mitigate the outcomes of the disease.
